# The epigenetic aging, obesity, and lifestyle

**DOI:** 10.3389/fcell.2022.985274

**Published:** 2022-09-13

**Authors:** Marica Franzago, Lucrezia Pilenzi, Sara Di Rado, Ester Vitacolonna, Liborio Stuppia

**Affiliations:** ^1^ Department of Medicine and Aging, School of Medicine and Health Sciences, G. d’Annunzio University, Chieti, Italy; ^2^ Center for Advanced Studies and Technology, G. d’Annunzio University, Chieti, Italy; ^3^ Department of Psychological Health and Territorial Sciences, School of Medicine and Health Sciences, G. d’Annunzio University, Chieti, Italy

**Keywords:** DNA methylation, DNAm age, epigenetics, obesity, lifestyle, BMI

## Abstract

The prevalence of obesity has dramatically increased worldwide over the past decades. Aging-related chronic conditions, such as type 2 diabetes and cardiovascular disease, are more prevalent in individuals with obesity, thus reducing their lifespan. Epigenetic clocks, the new metrics of biological age based on DNA methylation patterns, could be considered a reflection of the state of one’s health. Several environmental exposures and lifestyle factors can induce epigenetic aging accelerations, including obesity, thus leading to an increased risk of age-related diseases. The insight into the complex link between obesity and aging might have significant implications for the promotion of health and the mitigation of future disease risk. The present narrative review takes into account the interaction between epigenetic aging and obesity, suggesting that epigenome may be an intriguing target for age-related physiological changes and that its modification could influence aging and prolong a healthy lifespan. Therefore, we have focused on DNA methylation age as a clinical biomarker, as well as on the potential reversal of epigenetic age using a personalized diet- and lifestyle-based intervention.

## Introduction

Nowadays, the aging of the population represents a major social, economic, and public health challenge. Since aging, defined as a progressive loss of physiological integrity, is a key risk factor for many non-communicable diseases (NCD), including diabetes, cardiovascular, and metabolic diseases (Correa-Burrows et al., 2022). People of the same age may show differences in age-related functional decline as well as in their susceptibility to disease (Fransquet et al., 2019). Among the nine hallmarks which are considered the common denominators of aging at the molecular and cellular level, epigenetic changes, that are modifications of DNA or chromatin not altering the primary nucleotide sequence, can impact significantly on the aging process (López-Otín et al., 2013).

In this view, epigenetic marks, including DNA methylation, also known as “epigenetic clocks”, are emerging as a molecular tool for measuring biological aging (Tekola-Ayele, 2021) and, in this light, they could be considered as special “indicators” of the state of one’s health (Raj, 2018). In detail, the average of DNA methylation levels at multiple CpG sites (CpGs) is used to calculate the “epigenetic age”.

Many genes and pathways have been correlated with longevity regulation which could be possible epigenetic biomarkers of aging. The DNA epigenetic pattern changes during life. *ELOVL2* (fatty acid elongase 2) gene is the most extreme example of age-related hypermethylation, representing a bridge between the early stages of development and the aging process (Garagnani et al., 2012; Bacalini et al., 2017). This gene encodes for an enzyme that catalyzes one of the rate-limiting steps in the synthesis of long chain polyunsaturated fatty acids, which plays an important role in development (Gregory et al., 2011). *ELOVL2* is unmethylated in newborns, whereas its methylation levels significantly increase with age in different tissues, suggesting it as a kind of rheostat for aging-the more the gene is methylated, the older is the subject (Garagnani et al., 2012). Other possible genes proposed as biomarkers of aging include tumor suppressors (*COX7A1*, *LOX*, *RUNX3*, *TIG1*, *p16INK4A*, *RASSF1*, *DUSP22*) and genes involved in growth and development (IGF2, cFos), cell-cell adhesion (CDH1), metabolism (*SLC38A4*, *SLC22A18*, *MGC3207*, *ECRG4*, *ATP13A4*, *AGPAT2*, *LEP*), DNA repair (*MLH1*) and the control of signal transmission (*FZD1*, *FZD7*) (Christensen et al., 2009; Taormina and Mirisola, 2015). In addition, transcription factor-binding sites and promoters of genes involved in the regulation of gene expression, senescence, apoptosis and tumorigenesis are hypermethylated and silenced in a tissue-specific manner (Waki et al., 2003; Grönniger et al., 2010; Hernandez et al., 2011; Salminen et al., 2011). For example, the silencing of the *p16INK4a* gene (Keyes et al., 2007), which is a tumor suppressor and an aging-associated gene, is induced by hypermethylation of the transcription factor E2F-1 binding site within the gene promoter (Li and Tollefsbol, 2011).

Horvath et al. (Horvath, 2013) suggested that DNA methylation age (DNAmage) can evaluate the cumulative effect of an epigenetic maintenance system, identifying tissues that show evidence of accelerated age due to disease (Horvath, 2013). Epigenetic-age acceleration, defined as the discrepancy between the biological age measure and chronological age, have been used to examine the associations between epigenetic aging and several traits (Nannini et al., 2019). Overall, negative values of epigenetic-age acceleration (i.e., slower aging) are correlated with lifestyle factors, while positive values are associated with age-related health outcomes such as frailty, cancer, lung function and physical and cognitive fitness (Marioni et al., 2015; Breitling et al., 2016; Perna et al., 2016; Zheng et al., 2016).

Recently, it has been demonstrated that obesity accelerates epigenetic aging of metabolically active tissues including visceral adipose tissue and liver (Horvath et al., 2014; de Toro-Martín et al., 2019). Therefore, epigenetic-age acceleration has been correlated not only with obesity (Nevalainen et al., 2017), but also with physical fitness (Marioni et al., 2015) and stress (Zannas et al., 2015), albeit little is known about the link between epigenetic aging rates and lifestyle factors, including diet, alcohol abuse, and physical activity. The present narrative review considers the interaction between epigenetic clocks and age-related diseases, with particular emphasis on obesity. Some authors suggested the epigenome as an intriguing target for age-related physiological changes and its modification could prolong a healthy lifespan (López-Otín et al., 2013). Therefore, we have focused on DNA methylation age as clinical biomarker and on potential reversal of epigenetic age using a diet and lifestyle intervention.

## Obesity and aging

The prevalence of obesity has increased dramatically over the past decades. Obesity is associated with a higher risk of several age-related conditions and diseases, including cardiovascular disease (CVD), hypertension, type 2 diabetes mellitus (T2DM), and cancer (Pi-Sunyer, 2009). As a consequence, this condition shortens life expectancy by up to 20 years with the risk of premature death by 1.45 to 2.76-fold (Fontaine et al., 2003; Reuser et al., 2008). The mortality is lowest at a BMI of 22.5–25 kg/m2 range, with each 5 kg/m^2^ increase in the BMI associated with a 30% higher mortality, confirming the important role of BMI in the determination of lifespan (Prospective Studies Collaboration Whitlock et al., 2009). It is possible to suggest that obesity can accelerate aging, thus having a profound impact on human health. However, the relationship between aging and obesity is complex, involving cellular and molecular phenotypic signatures. Tam et al. (Tam et al., 2020) defined obesity and aging as “two sides of the same coin” since the pathology of the first condition parallels those of aging at multiple levels (Nannini et al., 2019).

**FIGURE 1:**
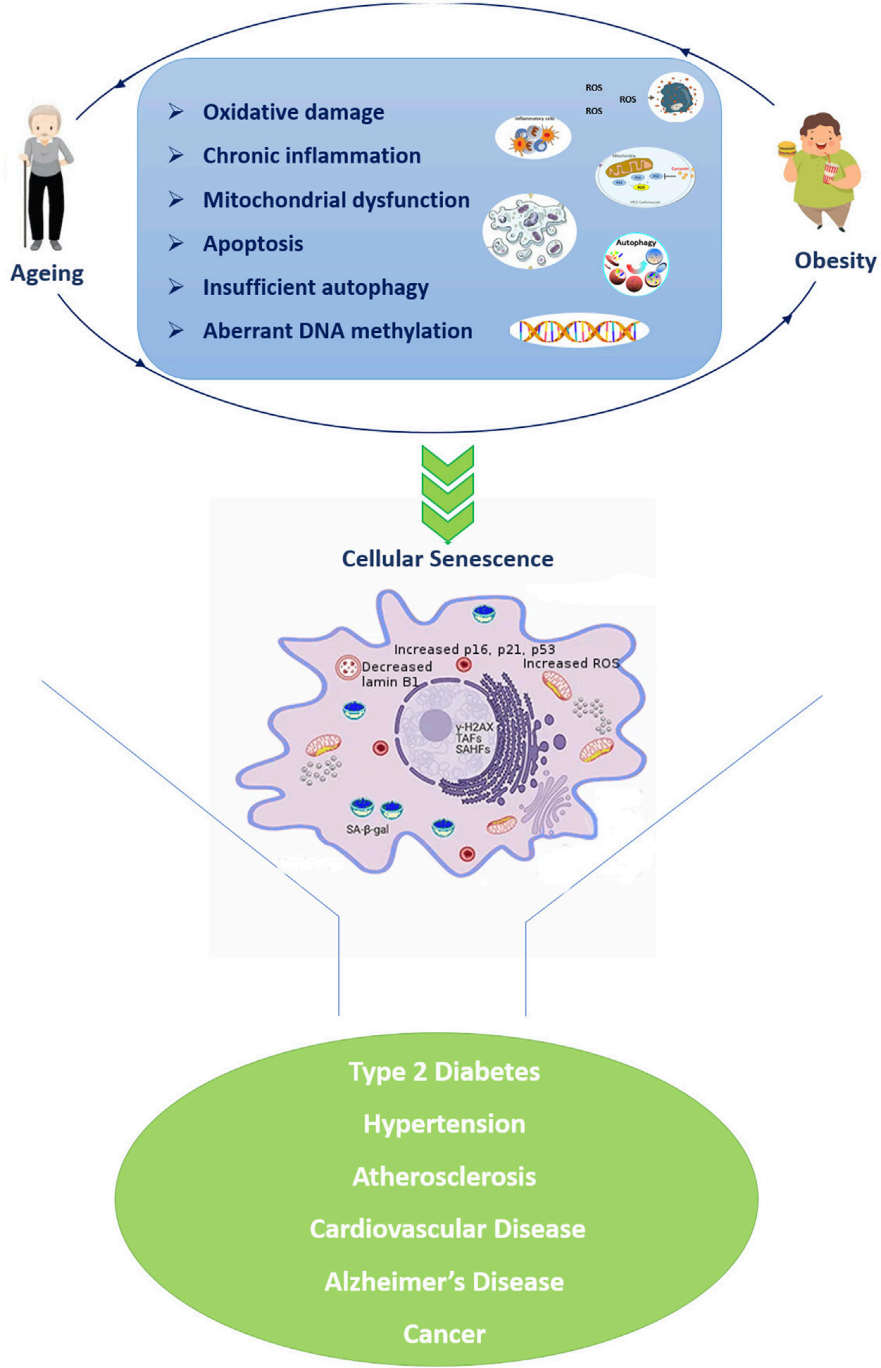
Similarities in cellular and molecular alterations of both ageing and obesity resulting in a vicious propagatory cycle which triggers the development of age-related diseases. The senescent cell was adapted from Narasimhan et al., 2021. Abbreviations: TAFs: telomere-associated DNA damage foci; SAHF: Senescence-associated heterochromatin foci; SA-β-gal: senescence-associated β-galactosidase.

In particular, the metabolic dysregulation correlated with obesity is similar to the one observed in aging and inflammation, oxidative stress, and impaired immune functions seem to be important mediators of this similarity (Santos and Sinha, 2021) (Figure 1). For example, the increased level of pro-inflammatory molecules is one of the major hallmarks of aging, in turn plays an important role in the adipose tissue enlargement. Obesity disrupts homeostatic resilience mechanisms which preserve physiological integrity, and the adipose tissue of obese subjects shows several of the hallmarks of aging, such as mitochondrial dysfunction, increased apoptosis, cellular senescence, insufficient autophagy, and chronic inflammation ((Pérez et al., 2016; Correa-Burrows et al., 2022). The excessive reactive oxygen species (ROS) formation triggers by chronic inflammatory cellular environment leading in oxidative stress and mitochondrial dysfunction, which results in further ROS formation and exacerbation of inflammatory processes. Overnutrition and obesity decrease autophagic flux resulting in excessive accumulation of malfunctioning organelles and misfolded proteins, which are common features of ageing. ROS formation compromises the mitochondrial DNA (mtDNA) integrity affecting the function of multiple tissues (Taylor and Turnbull, 2005), while the upregulation of pro-apoptotic genes and the caspase activation cascades resulting in the proteolytic cleavage of major structural proteins and the subsequent apoptosis (Yuzefovych et al., 2013). Moreover, DNA damage also triggers the upregulation of p21, p16ink4a playing important roles in DNA damage response. On the other hand, the chronic activation of DNA damage response and p53 induce senescence growth arrest (Freund et al., 2012). Chromatin reorganization triggers by the subsequent lamin B1 depletion in senescent cells, causes upregulation of senescence-associated secretory phenotypes (SASP) genes as well as packaging and silencing of proliferative genes promoting cellular senescence (Freund et al., 2012). Taken together, excessive ROS formation in obesity results in a broad range of cellular events which are key contributors to the onset of age-associated diseases. Moreover, obesity appears to influence telomere length as well as accelerate epigenetic clocks associated with aging (Buxton et al., 2011; Horvath, 2013; Clemente et al., 2019) as reviewed below.

## Epigenetic clocks (DNA methylation age)

Epigenetic mechanisms, such as DNA methylation, histone modification and microRNA (miRNA) regulation, can modulate gene expression without changing the DNA sequence. Environmental and lifestyle stressors disturb these epigenetic mechanisms, inducing long-term changes and potentially leading to disease in later life or even in the offspring, with a transgenerational effect, according to the developmental origin of health and disease (DOHaD) hypothesis (Stuppia et al., 2015). The aging process involves mainly DNA methylation, occurring at cytosines in a CpG dinucleotide (Lewis and Tollefsbol, 2017; Unnikrishnan et al., 2019) catalyzed by DNA methyltransferases (DNMTs). DNMTs family is responsible for establishing (DNMT3a and DNMT3b) and maintaining (DNMT1) the process of DNA methylation. Currently, there is a variability of reported findings showing the effects of age on the expression of the DNMTs. The life span of the heterozygous Dnmt1 knockout mice, which show a 33% decrease in DNMT1 expression, was similar to wild-type mice (Ray et al., 2006). On the other hand, the expression of the DNA methyltransferase Dnmt3a2 in the hippocampus were found to decrease with age (Cui and Xu, 2018) and the rescue of Dnmt3a2 levels restored cognitive functions (Oliveira et al., 2012). The expression of DNMT1 and DNMT3a were significantly higher in young dwarf mice compared with young wild-type mice; however, this difference was disappeared with age (Armstrong et al., 2014). Based on the results mentioned above, further studies are required to elucidate the expressional changes of DNMTs based on the aging.

A relationship between epigenetics and aging was first observed about 40 years ago in studies carried out in pink salmon and rat, which showed an overall decrease in DNA methylation with increasing age (Berdyshev et al., 1967; Vanyushin et al., 1973) This decrease has been subsequently also reported in humans. Drinkwater et al (Drinkwater et al., 1989) comparing DNAm in blood of young (average age 25 years) and older individuals (average age 75 years), showed that the elderly group had significantly reduced levels of 5 mC (Drinkwater et al., 1989). Several approaches for quantifying biological aging have been used over time, including telomere length and most recently epigenetic aging clocks. Telomeres, nucleoprotein structures containing tandem repeats of DNA (5′-TTAGGG-3′), are situated at the end of the chromosomes ((Biron-Shental et al., 2010)) and maintain their integrity and the stability of the genome, while preventing end-to-end chromosomal fusions as well (Lin et al., 2013). Shelterin, a complex of six telomere-specific proteins (TRF1, TRF2, TIN2, POT1, TPP1, and RAP1), is responsible for the protection of the genetic material, forming the so-called “t-loop” structure and together with the ribonucleoprotein reverse transcriptase, known as telomerase, modulate telomere length (de Lange, 2005). A proper function of the complex is required to prevent unwanted repair events and the trigger of the DNA damage response (Grun et al., 2018).

In the majority of somatic cells, telomeres become progressively shorter with each cell division, a process increased by oxidative stress and inflammation. As a result of telomere shortening, cell division slows down over the years, leading to accelerated cell senescence and a higher risk for developing age-related diseases such as CVD, T2DM, insulin resistance (Gardner et al., 2005), hypertension (Yang et al., 2009) and increased mortality (Levine et al., 2018). The association of leucocyte telomere length (LTL) and obesity has been also evidenced. The impact of high‐fat diet induced obesity and advancing age on the shelterin complex, and telomerase is unclear. Obesity and age influence the expression telomere shelterin complex and telomerase gene expression, altering telomere function in adipose tissue and thereby increasing inflammation and the risk of chronic disease (Bloom et al., 2020). Grun et al. (Grun et al., 2018) investigated TL from peripheral blood mononuclear cells in obese subjects analyzing the expression of shelterin genes and the plasma redox state. The authors showed dysregulation of the shelterin components that was explained by TRF1 upregulation, suggesting TRF1 as a major contributor for telomeres uncapping in the context of obesity (Grun et al., 2018). Some studies have suggested that weight loss *via* a structured multidisciplinary, personalized lifestyle intervention program of healthy diet and physical exercise might increase LTL, which, in turn, could potentially predict a better weight loss response to an intervention program in overweight and obese adolescents (Garcia-Calzon et al., 2014; Paltoglou et al., 2021).

Nowadays, epigenetic clocks play a crucial role in determining chronological age based on evaluation of hypo- and hyper-methylation changes in many regions of the genome (Fraga and Esteller, 2007; Zheng et al., 2016). The first-generation clocks used algorithms, based on DNAm as biomarker of aging (the Horvath and Hannum clocks (Hannum et al., 2013; Horvath, 2013), and were similar to a machine learning method that regresses chronological age on its selected set of the most informative CpG sites (CpGs) for age prediction. Thus, these models convert the methylation status of the selected CpGs into units of years, known as “DNAm age” (Horvath et al., 2014; Horvath and Raj, 2018). The Hannum estimator has been tested on blood-derived DNA and is based on DNA methylation at 71 CpGs selected from the Illumina 450k array, while the Horvath estimator (17) has been constructed across multiple tissues, including the blood data from Hannum et al., and comprises 353 CpGs that were present on the earlier generation Illumina 27k array. In recent years, ‘second generation clock’ models, such as DNAmGrimAge and the PhenoAge, have outperformed the ‘first-generation clocks’ in predicting longevity and the onset of many age-related pathological conditions and diseases (Levine et al., 2018). The DNAm-age estimator of Levine et al., defined PhenoAge clock, consists of 513 CpGs and is based on a measure of biological age comprising age and nine clinical biomarkers, including lymphocyte percentage, albumin, and glucose levels (Levine et al., 2018). Another multifactorial clock, DNAmGrimA, combines seven sets of CpGs, estimating the concentration of a different plasma protein, chronological age, sex and smoking habits (Lu et al., 2019).

More recently, a third-generation epigenetic clock, known as the Dunedin Pace of Aging methylation clock (DunedinPoAm) takes into account longitudinal change over time in 18 biomarkers (i.e. BMI, LTL and HDL cholesterol) of organ-system integrity among individuals who are all the same chronological age, recording how fast the subject is aging (Belsky et al., 2020) (Table1).

**TABLE 1:** Summary of first-generation (Hannum and Horvath), second (DNAm PhenoAge, GrimAge) and third-generation (DunedinPoAm) epigenetic clocks.

	Epigenetic clock (References)	Analysis of DNAm (#CpGs)	Surrogate markers (input)	Key features	Limits	Association with obesity	Association with other disease and clinical parameters
FIRST GENERATION	Horvath Horvath (2013)	353 CpGs	Naïve CD8^+^ T cells, exhausted CD8^+^ T, plasmablasts,CD4+ T cells, natural killer cells, monocytes and granulocytes	Chronological age. Methylation age across the lifespan. Estimation of both intrinsic and extrinsic epigenetic age	Estimations may be biased in older adults	For each 10 BMI units, 3.3 years increase was detected in epigenetic age Horvath et al. (2014). Association between obesity with EAA in adult liver tissue Horvath et al. (2014). Association between BMI with EAA in the whole blood of middle-aged adults Nevalainen et al. (2017). Association between obesity (BMI ≥30) with higher EAA (β = 0.43, CI: 0.24, 0.61, *p* < 0.001) Fiorito et al. (2019)	Cancer Dugue et al. (2018), Cellular senescence Lowe et al. (2016), Insulin level Quach et al. (2017) Levine et al. (2018), Pubertal development Binder et al. (2018), Menopause Levine et al. (2016)
	Hannum (Hannum et al. 2013)	71 CpGs	-	Chronological age. A more accurate prediction of life expectancy than Horvath clock	Tailored to adult blood samples and may lead to biased estimates in children and in nonblood tissues. Age estimations may be confounded by age-related changes in blood composition	Correlation between both EEAA and IEAA with BMI (*p* = 0.01and *p* = 0.05 respectively) mediated by associated metabolic syndrome Quachet al. (2017). Association between Obesity (BMI ≥30) with higher EAA (β = 0.20, CI: 0.05, 0.34, *p* < 0.05) Fiorito et al. (2019)	Blood pressure Quach et al. (2017), Cancer Dugue et al. (2018), Cholesterol, HDL Levine et al. (2018), Quach et al. (2017), Insulin level Quach et al. (2017), Levine et al. (2018)
SECOND GENERATION	PhenoAge Levine et al. (2018)	513 CpGs	Albumin, Creatinine,Glucose,C-reactive protein, Lymphocyte, Red cell volume, Alkaline phosphatase, White blood cell count	Improved predictive power over previous Horvath & Hannum clocks. Captures organismal age and the functional state of organs and tissues	Estimations may be biased in children and in nonblood tissues	Correlation between Obesity (BMI ≥30) with higher EAA (β = 1.01 CI: 0.74, 1.28, *p* < 0.001) Fiorito et al. (2019)	Cancer Dugue et al. (2018), CVD Levine et al. (2018), Coronary heart disease Levine et al. (2018), Cholesterol, HDL Levine et al. (2018) Quach et al. (2017), Insulin level Quach et al. (2017), Levine et al. (2018)
	GrimAge Lu et al. (2019)	1,030 CpGs	Age, sex, smoking, leptin,adrenomedullin,beta-2-microglobulin, cystatin C, growth differentiation factor 15, plasminogen activation inhibitor 1, tissue inhibitor metalloproteinase - 1	Currently best predictive epigenetic biomarker for lifespan and time to coronary heart disease (18 and 61%, respectively), more predictive than chronological age. Highlights healthy diet and educational attainment as predictors of biological age		Associations among BMI and insulin resistance with age acceleration Lundgren et al. (2021)	Cancer Dugue et al. (2018), Coronary heart disease Levine et al. (2018), Insulin level Quach et al. (2019), Levine et al. (2018), Menoapause Levine et al. (2016), Thurston et al. (2020)
THIRD GENERATION	DunedinPoAm (Belsky et al., 2020)	46 CpGs	HbA1C, Cardiorespiratory fitness, WHR, FEV1/FVC, FEV1, Mean arterial pressure, BMI, LTL, Creatinine clearance, Urea nitrogen, Lipoprotein, Triglycerides, Gum health, TC, White blood cell count, hsCRP HDL cholesterolApoB100/ApoA1	Designed to quantify the pace-of-aging. It built by measuring organ-system decline from young adulthood to midlife. Similarity of effect-sizes with GrimAge. Strongly correlated with a clinical-biomarker measure of biological age, with self-rated health, with functional test-performance and decline, and with morbidity and mortality as compared to other epigenetic clocks	Modest size of cohort. Need to establish cross-population validity	The CALERIE trial (2 years of prescribed 25% CR) provide proof-of-principle for DunedinPoAm as a single-time-point measure of a person’s pace of biological aging Belsky et al. (2020). CR altered DNAm at chronological-age associated CpGs in the direction of older age (*p* < 0.003 at 12- and 24-months follow-ups Ramaker et al. (2022). Correlation between BMI and DunedinPoAm in saliva DNA in socioeconomically disadvantaged children Raffington et al. (2021)	Hypertention,T2D, CVD, Chronic obstructive pulmonary disease, Chronic kidney disease, Cancer

DNAm: DNA, methylation; CALERIE: Comprehensive Assessment of Long term Effects of Reducing Intake of Energy, randomized clinical trial of caloric restriction; CR: caloric restriction; CVD: cardiovascular disease; Dunedin(*p*)ace(o)f(A)ging(m)ethylation:DunedinPoAm; TC: Total cholesterol; LTL: leukocyte telomere length; WHR; Waist-hip ratio; EAA: epigenetic age acceleration; EEAA: extrinsic epigenetic age acceleration; IEAA: intrinsic epigenetic age acceleration; T2D: Type-2, diabetes.

Epigenetic age acceleration (EAA) has been defined as the discrepancy between epigenetic age, estimated using the models described above, and chronological age. EAA can be divided into intrinsic acceleration (IEAA), which captures intrinsic cellular properties of the aging process and extrinsic acceleration (EEAA) which measures immune system aging (18). These measures have been widely used to evaluate the associations between epigenetic aging and numerous clinical traits and it has been evidenced that EEAA is affected by lifestyle (Nannini et al., 2019).

## Epigenetic clocks and obesity

Research on epigenetic biomarkers described more than a hundred of genes distributed over all chromosomes and associated with obesity and adiposity (Samblas et al., 2019).

BMI and obesity are associated with alterations of DNA methylation levels at several CpGs within metabolic genes including *HIF3A*, IGFBP3, SREBF1, TNF, T*RIM3* and *UBASH3A* as well as global methylation levels of Alu–elements genes (Houde et al., 2015; Samblas et al., 2019). However, some studies demonstrated that these obesity-related DNA methylation changes do not overlap those seen in aging-associated modifications (Marttila et al., 2015; Kananen et al., 2016), whereas other studies identified sites with a common effect in obesity and aging. In particular, it has been identified, by performing genome wide methylation analysis, 10 sites with an interaction effect between obesity and aging. In eight (*ADCY1*, *CXADR*, *KCNS2*, *LMX1B*, *FNDC4*, *NAT8L*, *AQPEP*, and *FBLIM1*) of the ten sites, the obese individuals displayed decreased level of methylation with age when compared to their lean counterparts (Almén et al., 2014). In contrast, the remaining two sites (RNH1 and NNAT) were hypermethylated with aging in the obese group compared to lean subjects (Almén et al., 2014). Leukocyte DNA methylation levels of several CpGs located at genes involved in longevity-regulating pathways have been evaluated in obesity, suggesting a role of DNA methylation in aging-related metabolic alterations. In details, the methylation levels of 58 CpGs located at genes involved in longevity-regulating pathways were associated with BMI. Fifteen of them were differentially methylated between younger and older individuals that exhibited at least one metabolic alteration. Of note, six of these CpGs, within *MTOR*, *ULK1*, *ADCY6*, *IGF1R*, *CREB5*, and *RELA*, were common to the metabolic traits, and *CREB5*, *RELA*, and *ULK1* were statistically correlated with age (Salas-Pérez et al., 2019).

Although the effects of DNA methylation on adipocyte aging process are yet to be fully elucidated, it has been demonstrated that some environmental factors and aging lead to modifications in methylation patterns in adipocytes. In particular, the senescence relevant genes hypomethylation such as p21, p16 as well as the telomerase reverse transcriptase (*TERT*)’s hypermethylation accelerate adipose progenitor cellular senescence and exhaustion (Qimuge et al., 2019; Pan et al., 2020). Moreover, an increase in methylation levels of the regulator regions in *Ppar-γ ADIPOQ* and *LEP* (Melzner et al., 2002; Fujiki et al., 2009; Kim et al., 2015) has been associated in white adipocyte glucose and lipid metabolism alterations, which further worsen insulin resistance, obesity, and inflammation. In addition, hypermethylation of *PRDM16* and the enhancer region of *UCP1* during cellular senescence, triggers to loss of beige adipose tissue and thermogenic properties of brown adipocytes (Yang et al., 2016; Foti and Brunetti, 2017). Of note, instead of focusing on single CpGs hypo- or hypermethylation patterns in single genes, the use of an epigenetic clock to study the relationship between obesity and the DNA methylation ages is based on the evaluation of the methylation status in many regions of the genome (Table 1). For example, Hovarth et al. (Horvath et al., 2014) showed 279 genes was underexpressed in older liver samples that are highly enriched with nuclear mitochondrial genes playing a role in oxidative phosphorylation and electron transport.

Although epigenetic clocks are recent metrics of biological age correlating with mortality risk (Marioni et al., 2018), it remains unclear whether epigenetic aging rates are associated with lifestyle factors such as diet, alcohol consumption, and BMI. For instance, the association between body composition and obesity with epigenetic clocks is not well understood. To date, it is unclear whether obesity is a driver or a consequence of epigenetic age acceleration (Tekola-Ayele, 2021). Some studies have investigated the obesity-related epigenetic aging in metabolically active tissues, confirming obesity-related epigenetic age acceleration in blood, liver, adipose, and buccal tissues (Horvath et al., 2014; Simpkin et al., 2017; de Toro-Martín et al., 2019).

Physical activity seems to attenuate the link between obesity and acceleration of the epigenetic clock: lower abilities in physical and mental fitness, measured by several markers (i.e. lung function, walking speed, grip strength and cognitive ability) have been associated with accelerated epigenetic aging (Marioni et al., 2015). Positive associations of body composition and physical activity level with multiple measures of epigenetic age acceleration have been recently found in blood from 2,758 non-Hispanic White women (Kresovich et al., 2021). Another previous study included data in blood tissue of more 4,100 postmenopausal female participants from the Women’s Health Initiative, as well as 402 male and female participants from the Italian cohort study. Interestingly, the research shows that diet, education, physical activity, low BMI, but not smoking habits, are associated with EEAA.

It is noteworthy that these findings confirmed the conventional benefits of eating a high plant diet with lean meats, moderate alcohol consumption, physical activity, and education, as well as the health risks of obesity and metabolic syndrome (Quach et al., 2017).

To characterize the significance of obesity in epigenetic aging, Nevalainen et al. (Nevalainen et al., 2017) studied the association between BMI and epigenetic age in three age groups: young adults, middle-aged, and nonagenarian individuals. Interestingly, the study confirmed the correlation between increased BMI and accelerated epigenetic aging in the blood cells of the middle-aged subjects, and this result was showed even when the BMI increased in adulthood (Nevalainen et al., 2017). More recent reporting (Mozhui et al., 2022) carried out in mouse models found that more rapid age-dependent changes in methylation in which higher weight gains at younger age are associated with higher epigenetic age acceleration later in life. In addition, the study showed that the effects of diet on epigenetic age acceleration was mediated by the changes in metabolic traits and weight, particularly, the latter had a stronger age-accelerating impact.

While the results described above confirm the link between accelerated epigenetic aging and obesity in adults, such evidence appears limited as far as it concerns children. Epigenetic age acceleration at birth and childhood has been associated with maternal habits, including smoking and alcohol consumption during the prenatal period (Simpkin et al., 2016). The link between epigenetic age acceleration with growth and development in early life remains unclear but the trajectory of the blood DNA methylome aging rate appears largely set before adulthood, remaining remarkably stable thereafter (Etzel et al., 2022). Recently, the association between accelerated epigenetic aging and BMI in children using multiple epigenetic clocks has been demonstrated (Gentilini et al., 2013). If the correlation between obesity and accelerated epigenetic aging begins in early life, epigenetic programming may offer potential and novel opportunity of intervention with implications for the promotion of health and the mitigation of future disease risk. In this context, since DNA methylation status changes with age, thus contributing to the development of age-related diseases, the study of centenarians’ offspring seems to be a valid approach to understand the role of epigenetics in the modulation of healthy aging. In this context, an age-related decrease in global DNA methylation and a delay of this process in centenarians’ offspring has been observed (Horvath et al., 2015). Moreover, a similar trend was reported by Horvath showing a lower intrinsic epigenetic ageing rate in peripheral blood mononuclear cells from Italian semi-supercentenarians and their offspring (Sae-Lee et al., 2018). Thus, the offspring of semi supercentenarians may be very informative when it comes to identifying epigenetic determinants of healthy aging and future relevant studies might be able to detect how to extend the benefits of successful aging.

## Lifestyle-reversal DNA methylation age

There is a growing interest to identifying therapeutic interventions modulating the mechanisms of aging or at least have the onset of age-related diseases slowed (Schork et al., 2022).

Among therapies targeting that slow the aging rate and mitigate damage to the body, have been proposed several approaches: 1) the seek to mimic the beneficial molecular effects of caloric restriction (Mahmoudi et al., 2019), 2) attempt to clear out senescent cells and the deleterious age-related debris that they secrete (Baker et al., 2016; Simpson et al., 2021), 3) the identification of circulating factors associated with healthy youth that could be infuse into older individuals (Conboy et al., 2013), 4) take advantage insights into stem cell biology and cellular rejuvenation (Olova et al., 2019; Browder et al., 2022).

On the other hand, the alteration of the environment and drug administration have been proposed as new approaches that could be utilized to extend life span due to their impact on epigenetic mechanisms and related metabolic activity (Longo et al., 2015). Some drugs, known as “epigenetic drugs” or “epi-drugs”, are chemical modifiers of epigenetic enzymes, specifically target DNMTs and are mainly used to treat cancer (Bates, 2020). On the other hand, based on a many similar mechanisms epigenetic regulation between aging and cancer (i.e., decline of heterochromatin DNA methylation, inhibitory histone modifications, increased DNA methylation in growth-related and tumor suppressor gene promoters), it could also be envisaged to apply epigenetic anticancer drugs to age-related diseases. This attractive strategy is being tested in ongoing clinical trials but demonstrate great promise about rejuvenation (Johnson et al., 2012) and may propose new possibilities to manage diseases.

Many studies support the hypothesis that dietary factors can reverse epigenetic alterations influencing the aging process: this newer approach could also offer compelling opportunities for the design of anti-aging treatments. Among dietary compounds, vitamins and polyphenols contribute to the production of the methyl donor S-adenosyl-l-methionine (SAM) and to the inhibition of DNA methyltransferases (DNMT) activity, respectively. By using publicly available Illumina Infinium 450K methylation datasets, it has been proven that dietary supplementation with folic acid + vitamin B12 as a methyl donor and flavanols as DNMT inhibitors can decrease epigenetic age and appear to be gender and *MTHFR* genotype-specific (Sae-Lee et al., 2018). It has been showed that calorie restriction and dietary rapamycin can slow molecular changes associated with an epigenetic clock in mice livers (Wang et al., 2017). Of note, caloric restriction decreased the age-related decline in DNMT1, DNMT3B, TET1, and TET3 gene expression in colon mucosa isolated from mice (Unnikrishnan et al., 2019). Previously, also Chouliaras et al. (Chouliaras et al., 2012) showed that the age-related increase in the level of DNMT3a protein in the hippocampus can be attenuated by dietary restriction.

However, although these results related to the reversal of epigenetic age might be exciting, it should be noted that while exercise can induce genome-wide changes in DNA methylation, the DNAm age of the adipose tissue seems not to be influenced (Rönn et al., 2013). These observations agree with previous studies showing that the bariatric surgery with significant weight loss, improved metabolic abnormalities but did not reverse epigenetic age acceleration in the liver of subjects within a 9-month period (Horvath et al., 2014). A 24- month randomized factorial intervention trial carried out in healthy postmenopausal women provided strong evidence of a causal relationship between lifestyle and healthy aging-related epigenetic mechanisms. The effect of a dietary intervention reflected a slowing of the DNAmGrimAge clock, and the increase of physical activity led to a reduction of stochastic epigenetic mutations, which have been proposed as a complementary DNAm-based biomarker of healthy aging (Fiorito et al., 2021). Taken together, the key issues remain whether: 1) these tissue-specific effects are reflected epigenetic aging rates in the same way as for other tissues; 2) the reversal of epigenetic ageing is effectively possible through lifestyle improvement.

Some findings shed light on the potential utility of epigenetic clocks for studying key traits and changes during critically important period of life. In women menopause represents a crucial turning point, because it not only marks the end of the fertile age, but also accelerates the general aging processes, with significant repercussions on health as a whole. Addressing sexual aging adequately –(particularly with the healthy lifestyle) can prevent several issues. Aging and reproduction are intrinsically linked although future work examining the relationship between epigenetic clocks and reproductive health is warranted. The effect of reproduction on epigenetic clocks may be tied to modifications in immune cell composition of blood and to hormonal changes (Ryan, 2021). Postmenopausal women with a late onset of menopause are epigenetically younger than women with an early onset of menopause (Levine et al., 2016). In addition, women who have undergone surgical menopause (bilateral oophorectomy) showed accelerated epigenetic age, appearing to be decelerated with menopausal hormone therapies (Levine et al., 2016). Similarly, severe vasomotor symptoms (hot flashes) among older women were associated with higher DNAm PhenoAge as well as late-occurring hot flashes were linked to accelerated DNAm PhenoAge and DNAm GrimAge (Thurston et al., 2020).

## Discussion

The rapid rise of obesity causes for concern, and it is attributed mainly to the lifestyle and diet adopted recently. However, both genetic variants and epigenetic changes may affect some subjects who are more susceptible to this obesogenic environment, leading to obesity and its complications (Franzago et al., 2020). Since obesity is linked to aging, epigenetic processes might play a role in healthy human aging. There is great interest for epigenetic markers with regard to their future beneficial application in the promotion for healt. Although lifestyle factors such as diet, alcohol consumption, and physical activity are connected to health-related outcomes, the literature on the direct influence of these factors on molecular aging rates is only at its early stage. Clarifying the molecular determinants of healthy aging and longevity would provide a major contribution to current research. The promising ultimate tools for investigating the relationship between lifestyle and aging are the molecular biomarkers known as the “epigenetic clocks”, which are based on the quantification of DNA methylation levels, apart from LTL.

The interest of the epigenetic mechanisms is due to their reversibility as well as their possible transgenerational effects (Franzago et al., 2019), which can be detected by the predictor of epigenetic age. The above has the potential to elucidate epigenetic inheritance and the effects caused by the environment and lifestyle that can slow down or accelerate epigenetic aging. In particular, the recent attention for epigenetic clocks as a biomarker of ageing is due to a 1) their application to several human tissues, 2) their accurate measurement of chronological age (Gibbs, 2014), 3) their independent predictive value for all-cause mortality in later life (Marioni et al., 2015), 4) their association with cognitive and physical factors in the elderly (Marioni et al., 2015) and 5) their usefulness in identifying accelerated ageing effects due to obesity and other conditions (Lowe et al., 2016; Binder et al., 2018; Dugué et al., 2018; Fiorito et al., 2019; Lundgren et al., 2021; Narasimhan et al., 2021; Ramaker et al., 2022)

**FIGURE 2:**
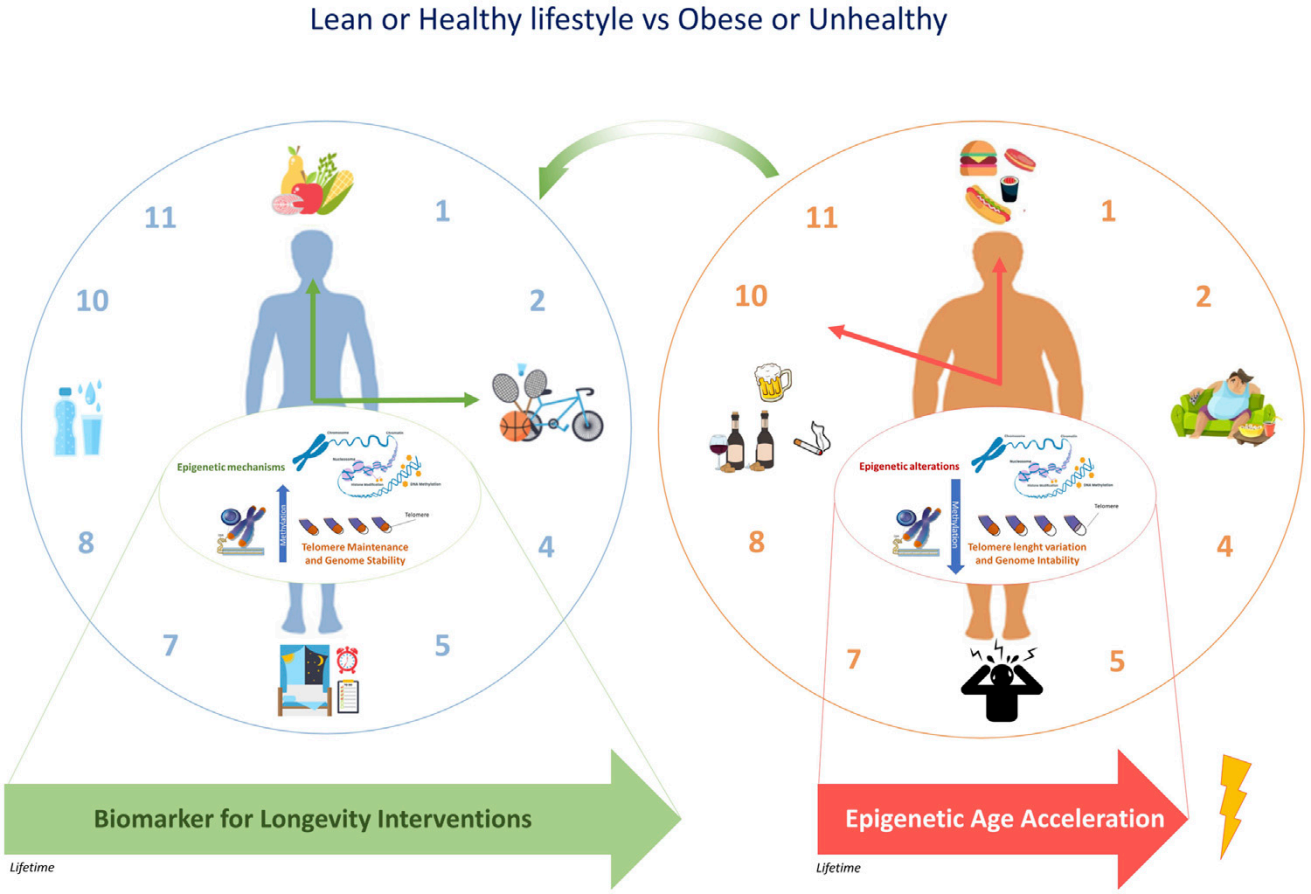
The epigenetic clock and telomere length are associated with chronological age. The epigenetic modifications induced by an unhealthy lifestyle can accelerate epigenetic aging. Due to the potential reversal of epigenetic aging, some targeted population groups, such as obese subjects might respond to changes in lifestyle.

Overall, epigenetic clocks are considered accurate biomarkers of the wider process of epigenetic remodeling occurring in different tissues during aging and might provide optimal strategies to improve the health of the population, especially if targeting specific groups, such as obese subjects (Figure 2). Recent literature supported that the modifications in epigenetic acceleration are correlated with change in BMI, suggesting that epigenetic aging may respond to changes in lifestyle, at least with respect to change in obesity (Quach et al., 2017). This perspective leads the way towards considering epigenetic biomarkers in dietary intervention studies in the future. Further research is warranted in order to gain further insight into the physiological relevance of epigenetic aging and to its role in the onset of lifestyle-related diseases such as obesity.
